# Indigenizing and co-producing the ACGME anesthesiology milestone in Taiwan: a Delphi study and subgroup analysis

**DOI:** 10.1186/s12909-024-05081-2

**Published:** 2024-02-19

**Authors:** Enoch Yi-No Kang, Kuan-Yu Chi, Faith Liao, Chih-Chung Liu, Chih-Peng Lin, Ta-Liang Chen, Pedro Tanaka, Chien-Yu Chen

**Affiliations:** 1 Department of Anesthesiology, School of Medicine, College of Medicine, Taipei Medical University, Taipei, Taiwan; 2 Institute of Health Policy & Management, College of Public Health, National Taiwan University, Taipei, Taiwan; 3 Department of Education and Humanities in Medicine, School of Medicine, College of Medicine, Taipei Medical University, Taipei, Taiwan; 4 Department of Medicine, Jacobi Medical Center, Bronx, NY USA; 5 Albert Einstein College of Medicine, Bronx, NY USA; 6 Department of Education, Taipei Medical University Hospital, Taipei, Taiwan; 7 Department of Anesthesiology, Taipei Medical University Hospital, Taipei, Taiwan; 8 Department of Anesthesiology, National Taiwan University Hospital, National Taiwan University College of Medicine, Taipei, Taiwan; 9 Department of Anesthesiology, Wan Fang Hospital, Taipei Medical University, Taipei, Taiwan; 10 Department of Anesthesia, Stanford University Medical School, Palo Alto, CA USA

**Keywords:** Milestone, Competency-based medical education, Resident training, Anesthesiologist, Delphi

## Abstract

**Background:**

To implement the ACGME Anesthesiology Milestone Project in a non-North American context, a process of indigenization is essential. In this study, we aim to explore the differences in perspective toward the anesthesiology competencies among residents and junior and senior visiting staff members and co-produce a preliminary framework for the following nation-wide survey in Taiwan.

**Methods:**

The expert committee translation and Delphi technique were adopted to co-construct an indigenized draft of milestones. Descriptive analysis, chi-square testing, Pearson correlation testing, and repeated-measures analysis of variance in the general linear model were employed to calculate the *F* values and mean differences (MDs).

**Results:**

The translation committee included three experts and the consensus panel recruited 37 participants from four hospitals in Taiwan: 9 residents, 13 junior visiting staff members (JVSs), and 15 senior visiting staff members (SVSs). The consensus on the content of the 285 milestones was achieved after 271 minor and 6 major modifications in 3 rounds of the Delphi survey. Moreover, JVSs were more concerned regarding patient care than were both residents (MD = − 0.095, *P <* 0.001) and SVSs (MD = 0.075, *P <* 0.001). Residents were more concerned regarding practice-based learning improvement than were JVSs (MD = 0.081; *P <* 0.01); they also acknowledged professionalism more than JVSs (MD = 0.072; *P <* 0.05) and SVSs (MD = 0.12; *P <* 0.01). Finally, SVSs graded interpersonal and communication skills lower than both residents (MD = 0.068; *P <* 0.05) and JVSs (MD = 0.065; *P <* 0.05) did.

**Conclusions:**

Most ACGME anesthesiology milestones are applicable and feasible in Taiwan. Incorporating residents’ perspectives may bring insight and facilitate shared understanding to a new educational implementation. This study helped Taiwan generate a well-informed and indigenized draft of a competency-based framework for the following nation-wide Delphi survey.

**Supplementary Information:**

The online version contains supplementary material available at 10.1186/s12909-024-05081-2.

## Highlight

1. Most ACGME anesthesiology milestones are applicable in Taiwan.

2. Experienced anesthesiologists achieved consensus faster than young practitioners.

3. Residents mirrored milestone competencies through participation.

4. Experience status affected the weight that anesthesiologists gave to milestone competencies.

**Supplementary Information:**

The online version contains supplementary material available at 10.1186/s12909-024-05081-2.

## Introduction

With the continuous development of science and technology, the format of residency training has encountered a paradigm shift from time-based medical education to competency-based medical education (CBME) over the last two decades [1]. This shift has had an enormous impact on resident training programs, reflecting the efforts of the Accreditation Council for Graduate Medical Education (ACGME) to establish CBME for all physicians. For instance, milestones competencies, known as ACGME reporting milestones, include patient care (PC), medical knowledge (MK), systems-based practice (SBP), practice-based learning and improvement (PBLI), professionalism (PROF), and interpersonal and communication skills (ICS), which are considered the criteria to indicate well-developed medical professionals. Notably, the ACGME offers more than 100 specialties and subspecialties, including the Anesthesiology Milestone Project (a workable CBME framework), all of which facilitate learners’ progress from the novice to the expert level with the expected proficiency [2]. Only by explicitly articulating residents’ developmental milestones and outcome competencies could the competency-based teaching, learning, and assessment strategies be developed and performed sequentially and deliberately [3].

The Anesthesiology Milestone Project, initiated conjointly by the ACGME and the American Board of Anesthesiology, has been officially implemented in all the residency training programs in the United States since 2015 [4]. To adopt the CBME conceptual model crossculturally in a foreign clinical and educational system, the indigenizing process such as mixed method exploratory triangulation [5, 6], back-translation [7], or two-step validation [8, 9] were borrowed. Indigenization, in the context of document translation, refers to the process of adapting a document to the cultural and linguistic context of an “indigenous” community. It goes beyond a simple translation of the content and involves considering the cultural nuances, such as professional ethos and hidden curriculum. This ensures that the translated document is culturally appropriate, respectful, and resonates with the target audience. Three issues have been addressed in our indigenization process. First, since the ACGME milestone is reported in English, it needs to be carefully translated and interpreted to the common “language” used and understood by the local practitioners [10]. Expert committee translation may ensure content accuracy and prevent cultural loss in the local context during CBME adoption [11]. Second, the co-production model of healthcare and its education, which values patients’ and learners’ engagement, was urged nowadays to facilitate sustainability and desired outcomes [5, 6, 8, 12]. Such a conceptual model could be applied to a consensus study by deliberately recruiting different categories of key stakeholders [8, 13, 14]. Considering the junior members’ perspectives is essential to meeting the learners’ needs. Co-production model has been proposed as an appropriate approach to develop trainees’ competencies, and ACGME also encourages attendings to guide residents using co-production model [15, 16]. A well-structured medical education program, which requires co-planning, co-executing, and co-producing the learning experience between the trainers and trainees collaboratively, may provide a substantial learning outcome and increase health care quality [12, 15, 16].

Third, the Delphi technique, considered a consensus method, has been used to establish consensus, develop concepts, and formulate future research directions across a range of subjects [17]. The opinions of panelists are iteratively proposed, collected, and analyzed by using this method until any disagreement is resolved [18]. This approach is primarily used for curriculum development, policy-setting, criterion-setting, and goal-setting and thus has been widely applied in medical education to develop curricula [19–22] and assess learning outcomes [23, 24]. Furthermore, subgroup analysis could be employed in a Delphi survey to unfold the differences between panels [13, 25, 26]. Bringing in such contextual insight may facilitate trainer–trainee cooperation for designing, developing, and delivering medical education and improving the overall learning process and outcome.

This study is a preliminary work of a two-year project (funded by the Taiwan Ministry of Science and Technology, MOST 105-2511-S-038-003), piloted in four teaching hospitals for preparing the following nation-wide Delphi survey [27]. The primary objective of this study is to review the relevance and evaluability of the ACGME 25 sub-competencies and their 285 milestones by all stakeholders, including senior visiting staff members (SVSs), junior visiting staff members (JVSs), and residents. The secondary research objective is to compare the differences between generations and investigate the trainers’ and trainees’ conceptual diversity during the multi-step consensus development process.

## Methods

The expert committee was employed to ensure the translation quality before the survey. The Delphi technique was applied for adapting and co-producing the ACGME milestones. The qualitative feedback, achievement of consensus, and trend consistencies were analyzed, presented, and replied to participants during the multiple rounds of the Delphi survey. This study was approved by the Taipei Medical University–Joint Institutional Review Board (TMU-JIRB) with serial number N201604060, and written informed consent was obtained from all participants.

### Expert committee translation and questionnaire design

**Fig. 1:**
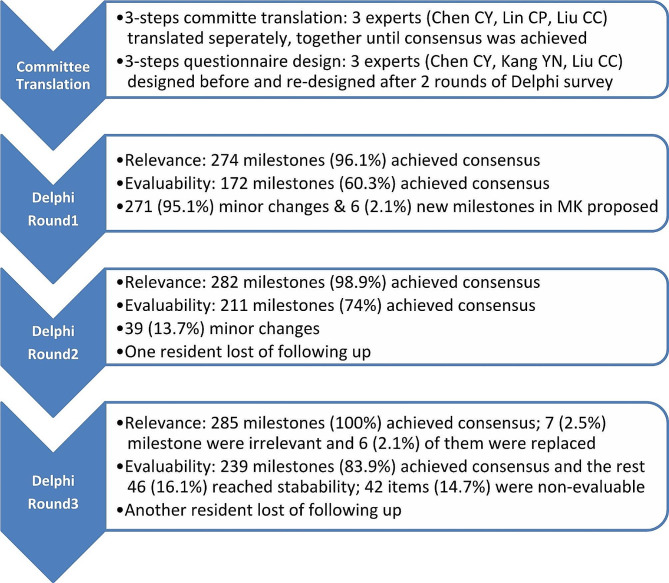
Flow chart of study steps and the primary outcomes

The strategy of expert committee translation [11] was employed to ensure content accuracy from English to Traditional Chinese. Three experts (Chen CY, Lin CP, Liu CC) who are bilingually knowledgeable in anesthesiology and medical education were recruited to work separately (1st step) and together (2nd step) until consensus was achieved (3rd step). After the first Chinese draft of the ACGME anesthesiology milestone was developed, three experts (Chen CY, Kang YN, Liu CC) co-designed an online Delphi questionnaire. We used the Google Docs platform to investigate the relevance and evaluability of all 285 milestones under 25 sub-competencies. The other two questionnaire rounds were re-designed by the same team after each Delphi round. Figure 1 illustrates the research steps of this study.

No specific structure was established prior to data analysis to facilitate the natural development of a theoretical framework from the comments, thus mitigating potential bias stemming from the subjective inclinations and opinions of the researchers. The analytical process comprised three sequential steps, leveraging grounded theory, an inductive methodology that provides systematic guidance for the collection, synthesis, analysis, and conceptualization of qualitative data, ultimately aiming to construct theory [28]. In practice, experts were asked to provide their opinions on any inappropriate items in the Mandarin version, and researchers categorized those opinions into sub-themes following a comprehensive review. They subsequently grouped similar sub-themes together to form main themes.

### Delphi panels

The sample size required for the Delphi technique typically ranges from 15 to 30 experts from the same discipline [29–31]. We targeted to recruit three panels (i.e., R, JVS, SVS) to incorporate various perspectives and further compare the panels’ differences. To collect useful opinions from the Delphi survey, our inclusion criteria were: (a) anesthesiologists work in teaching hospitals or medical centers during our study, and (b) they received relevant lectures or workshops about the ACGME milestone. We did not recruit year-one residents because they had no whole picture of anesthesia in clinical practice. Therefore, we invited 40 anesthesiologists to participate; of them, the 39 who agreed to participate included 15 SVSs, 13 JVSs, and 11 residents from two medical centers and two teaching hospitals. Two residents (5.1%) lost of following up in round 2 and 3, respectively. The SVSs and JVSs had an experience of ≥ 10 and < 10 years, respectively.

### Data collection and analysis

Delphi survey was used for qualitative and quantitative data collection, whereby scores and interpretations of individual ACGME anesthesiology milestone items were explored separately. Moreover, this study examined the differences in applicable scores of the six domains and five levels among three anesthesiologist experience statuses in the final round (round 3) of the Delphi survey. The Delphi survey used a 4-point Likert scale, ranging from 1 (*disagree*) and 4 (*agree*). Research has shown that using fewer scale points can lead to higher reliability [32], with four to seven points commonly used in studies [33]. Additionally, evidence suggests that cultural differences play a role in survey studies [34, 35], and in our cultural context, a 4-point scale was chosen for our expert survey. Because social expectations and hierarchical pressure in their hospital may have led to bias among our anesthesiologist experts, online questionnaire and anonymity were deliberately employed. A research assistant was trained to manage the Delphi survey by using a standard approach; this involved the construction of an anonymous Google questionnaire for the Delphi survey. After completing qualitative and quantitative data collection, the research assistant sent a summary of each survey round to all participants; moreover, this summary report was completely anonymized. All qualitative comments were carefully reviewed and taken into consideration for co-producing an indigenized anesthesiology milestone. The qualitative feedback, achievement of consensus, and trend consistencies (i.e., consistency between different rounds) were analyzed, presented, and replied to participants during the multiple rounds of the Delphi survey.

### Subgroup analysis

The Delphi method facilitated the forecasting process of negotiating nonconsensus items among the 37 anesthesiologists’ perspectives. We judged nonconsensus by using interquartile ranges (IQR) with the relevant methodology. Because an IQR demonstrates dispersion from the median and consists of the middle 50% of the cases, an IQR of ≤ 1 indicates that > 50% of the expert responses fall within 1 point [36]. By contrast, if an IQR of > 1 is obtained in each study round, the nonconsensus items are excluded from or revised in each subsequent round. The results of each round were presented using a 2 × 3 contingency table reporting the results of the chi-square test of independence and displaying Cramer’s V. Trend consistencies were also examined using Pearson correlation testing. Multiple pairwise comparisons were performed using the general linear model (GLM) for the differences in the applicable scores of the six domains and five levels among the three experience statuses. The results of the GLM analyses are presented as *F* values, mean differences (MDs), and standard errors (SEs), all with their corresponding 95% confidence intervals (CIs).

## Results

After three rounds of Delphi surveys, all 285 milestones have achieved either consensus or stability. 274 (96.1%) and 172 (60.3%) milestones were regarded as relevant and evaluable, respectively, in round one (Fig. 1). Seven milestones did not reach consensus in the first round, and we raised three of them as examples as follows:

After adding six newly proposed milestones, another 8 and 39 milestones achieved consensus in round two. 2.5% (*n* = 7) and 14.7% (*n* = 42) of the anesthesiology milestones were regarded as *irrelevant* and *non-evaluable*, respectively. 271 (95.1%) milestones were revised or rephrased without changing their original meaning, whereas 6 (2.1%) milestones in medical knowledge have been replaced because all of them are the exams held in the US. The qualitative comments (*n* = 1145) were collected, analyzed, and categorized into four main themes: *definition, relevance, evaluability*, and *others*; and their sub-themes and representative comments were presented in Table 1. The qualitative analysis for theme development provided contextual meanings and practical insights into our milestone indigenization process. For instance, the comments in the sub-theme of ***irrelevant*** offered the rationale why a milestone should be deleted, and those in the sub-theme of ***translation*** provided a more accurate wording in Mandarin to rephrase a milestone.

**Table 1:** Themes, sub-themes, and representative comments

Theme/sub-theme	Representative comments
**1. Definition (*n*** **=** **226)**	
1.1 Translation	“Able to integrate evidence-based medicine (EBM)” changed to “receive and apply EBM.” “Can adjust quickly” changed to “Know when to consult others.”
1.2 Critics	The medical records can show the “medical decision-making process and the promotion of patient care,” but also “succinct”; there is a certain degree of conflict and contradiction with each other. What is meant by an “ideal role model,” just like the previous “master model,” is difficult to quantify, empty and vague, and difficult to evaluate. Without specific instructions, it is difficult to imagine the ability to “balance.”
1.3 Key work definition	Development of organizational policies and procedures for “impaired physicians”: Is there such a thing? “Conditional,” “complex,” and “challenging” all need to be defined first. The details of the anesthesia care goals set by the CCC are unclear.
**2. Relevance (*n*** **=** **354)**	
3.1 Highly relevant	In a modern society where medical disputes are increasing, residents must learn this during the training process. Troubleshooting should not just be the job of an anesthesia technician. Residents should also be able to troubleshoot problems in emergencies.
3.2 Currently irrelevant	The existing mechanism does lack the evaluation indicators of long-term results. Suggest to delete it. Pain injections, scar injections, lumbar epidural steroid injections, and intravenous regional blockades are the items not easy to have opportunities to practice. It is an appropriate indicator for the attending but not practical for the resident.
3.3 Irrelevant	As far as R1 is concerned, some subspecialty topics have exceeded their capabilities We don’t have this kind of resources and power ourselves; how do we evaluate the subjects?
**3. Evaluability (*n*** **=** **412)**	
3.1 Assessment tool	We can consider breaking up those milestones and incorporated them into the written test, oral test, simulation. We can evaluate a part of it through reflective writing and discussion. However, I suspect whether a clinical practice can be easily assessed through reflection. We can apply the ISBAR checklist to the education and evaluation of hand-over.
3.2 Policy and resource	Physicians’ dedication to teaching might need to reduce their clinical workload. There should be a particular department or unit to take up this job.
3.3 Implementation strategy	Evaluation speed or contingency results? I recommend evaluating separately and use simulation to add the “time” factor to the evaluation process. Try to reflect on clinical practice with EBM methods (not necessarily requiring conclusions or alteration.)
**4. Others (*n*** **=** **153)**	
4.1 Learning resource	Each hospital has a different system, but you should know what resources you have in your hospital. Need equipment support or relevant training in the department. There was no training course in the past. I recommend holding this training course.
4.2 Competency level	It is a bit high for Level 2 Level 1 trainees will not handle disagreements under general anesthesia.
4.3 Socio-cultural diversity	Different medical institutions handle medical disputes in different ways. Some institutions do not recommend that doctors individually report medical errors. In this case, how to evaluate it? Such an intervention might not be suitable in a different national context!!
4.4 Faculty development	The younger generation nowadays is even better at mastering ultrasound than senior attending physicians. How do we know attending physicians care capable of assessing residents? That is the problem I concern most. Not every training center has a group of professional pain specialists with relevant guidance capabilities.

We further performed chi-squared test for subgroup analysis on the panels of residents, JVSs, and SVSs separately (Table 2). In the final round of this separate Delphi analysis, all experts achieved significant consensus regardless of their experience status (*P =* 0.059). However, in round 1 of this analysis, residents, JVSs, and SVSs had 41 (14.44%), 22 (7.72%), and 6 (2.11%) nonconsensus items, respectively (*P <* 0.01). To address the discrepancy, we conducted round 2. Then, SVSs attained 100% consensus in round 2 with the lowest number of nonconsensus items, whereas JVSs and residents had one and five nonconsensus items, respectively. The difference was significant (*P =* 0.013) for one nonstable nonconsensus item out of the three nonconsensus items among JVSs. Consequently, we performed round 3 to minimize nonstable nonconsensus. Though there is still one nonconsensus item, which was a stable event, all statuses are comparable for the consensus throughout round 3.

**Table 2:** Differences in non-consensus items among levels of anesthesiologists in each round of Delphi study

Domain	R	JVS	SVS	χ^2^	V	p
**Round 1**				29.20	0.185	< 0.001
NSNC	41 ^a^ (14.44%)	22 ^b^ (7.72%)	6 ^c^ (2.11%)			
Consensus	244 ^a^ (85.56%)	263 ^b^ (92.28%)	279^c^(97.89%)			
**Round 2**				12.67	0.122	0.013
NSNC	5 ^a^ (1.76%)	2^a^ (0.7%)	0 ^a^ (0%)			
SNC	5 ^a^ (1.76%)	1 ^a^ (0.35%)	0 ^a^ (0%)			
Consensus	273 ^a^ (96.48%)	280 ^a,b^ (98.95%)	283 ^b^ (100%)			
**Round 3**				9.07	0.103	0.059
NSNC	0 ^a^ (0%)	1 ^a^ (0.35%)	0 ^a^ (0%)			
SNC	5 ^a^ (1.76%)	1 ^a^ (0.35%)	0 ^a^ (0%)			
Consensus	279 ^a^ (98.24%)	282 ^a^ (99.3%)	284 ^a^ (100%)			

Note. Each subscript letter denotes a subset of ID categories whose column proportions do not differ significantly from each other at the 0.05 level. *V*, Cramer’s V. SNC, stable non-consensus. NSNC, non-stable non-consensus

During all three rounds above, the correlation coefficients between rounds increased round by round, consistently and significantly (*P <* 0.001). Among residents, JVSs and SVSs, the correlation coefficients increased from 0.965, 0.896, and 0.962, respectively, for rounds 1 and 2 to 0.984, 0.998, and 1.00, respectively, for rounds 2 and 3. Notably, SVSs attained a positive correlation at the end of all rounds; in other words, all 15 SVSs reached consensus for each item (Table 3). By contrast, the correlation coefficients between experience statuses for each round demonstrated a rather decremental trend with significantly positive correlation. For rounds 1, 2, and 3, the resident–JVS correlation coefficients were 0.834, 0.735, and 0.724, respectively; similarly, resident–SVS correlation coefficients were 0.817 and 0.799 for the rounds 1 and 2, respectively.

**Table 3:** Correlation between levels of anesthesiologists in medical hierarchy in each round of Delphi study

	Round 1			Round 2			Round 3		
Status	R	JVS	SVS	R	JVS	SVS	R	JVS	SVS
R	--	**0.834** ^***^	**0.817** ^***^	*0.965* ^***a^	**0.735** ^***^	**0.820** ^***^	*0.984* ^***b^	**0.724** ^***^	**0.799** ^***^
JVS	--	--	**0.870** ^***^	--	*0.896* ^***a^	**0.784** ^***^	--	*0.998* ^***b^	**0.788** ^***^
SVS	--	--	--	--	--	*0.962* ^***a^	--	--	*1.00* ^***b^

Note. *** *p*-value < 0.001. Bold is the correlation coefficients in the same round. Italic is correlation coefficients between round to round. (a) Correlation coefficients between round 1 and round 2. (b) Correlation coefficients between round 2 and round 3

Regarding the six core competencies of the ACGME milestones, the pair comparisons among the experience statuses in round 3 are presented in Table 4. The difference in every pair of comparison was small for the MK and SBP domains, with four pairs presenting marginal difference: resident–JVS in MK (MD = − 0.37; 95% CI = − 0.79, 0.04; *P <* 0.10), JVS–SVS in SBP (MD = − 0.06; 95% CI = − 0.14, 0.01), resident–SVS in PBLI (MD = 0.072; 95% CI = 0.00, 0.15), and JVS–SVS in PROF (MD = 0.047; 95% CI = − 0.01, 0.10; all *P <* 0.10). By contrast, for the PC domain, values of JVSs were significantly higher than those of both residents (MD = − 0.095; 95% CI = − 0.15, − 0.04) and SVSs (MD = 0.075; 95% CI = 0.04, 0.11; both *P <* 0.001). Regarding PBLI, values of residents were significantly higher than those of JVSs (MD = 0.081; 95% CI = 0.03, 0.14; *P <* 0.01) and were less significantly higher than those of SVSs (MD = 0.072; 95% CI = 0.00, 0.15; *P <* 0.1). Moreover, residents acknowledged PROF more than either JVSs (MD = 0.072; 95% CI = 0.02, 0.13; *P <* 0.05) or SVSs (MD = 0.12; 95% CI = 0.04, 0.2; *P <* 0.01) did. Finally, SVSs graded ICS significantly lower than both residents (MD = 0.068; 95% CI = 0.01, 0.13; *P* < 0.05) and JVSs (MD = 0.065; 95% CI = 0.01, 0.12; *P* < 0.05) did.

**Table 4:** Multiple comparisons between statuses in the final Delphi survey round on six domains

	R vs. JVS			R vs. SVS			JVS vs. SVS		
Domain	*MD*	*SE*	*95%CI*	*M*	*SE*	*95%CI*	*M*	*SE*	*95%CI*
**PC**	− 0.095^***^	0.026	[-0.15, -0.04]	-0.01	0.023	[-0.07, 0.03]	0.075^***^	0.019	[0.04, 0.11]
**MK**	-0.37^†^	0.191	[-0.79, 0.04]	-0.02	0.112	[-0.27, 0.22]	0.352	0.196	[-0.08, 0.78]
**SBP**	0.045	0.045	[-0.05, 0.14]	-0.01	0.035	[-0.09, 0.05]	-0.06^†^	0.035	[-0.14, 0.01]
**PBLI**	0.081^**^	0.027	[0.03, 0.14]	0.072^†^	0.037	[0.00, 0.15]	-0.00	0.032	[-0.07, 0.06]
**PROF**	0.072^*^	0.028	[0.02, 0.13]	0.120^**^	0.039	[0.04, 0.2]	0.047^†^	0.027	[-0.01, 0.10]
**ICS**	0.002	0.029	[-0.06, 0.06]	0.068^*^	0.030	[0.01, 0.13]	0.065^*^	0.028	[0.01, 0.12]

Note. Adjustment for multiple comparisons: Least Significant Difference. † *p*-value < 0.10; * *p*-value < 0.05; ** *p*-value < 0.01; *** *p*-value < 0.001

Regarding the five levels of the six domains of ACGME milestones (Table 5), the pair comparisons between experience statuses revealed almost no effect at levels 1 and 3, except for marginal differences in the resident–JVS pair at level 1 (MD = − 0.04; 95%CI = − 0.10, 0.00) and the resident–SVS pair at level 3 (MD = 0.057; 95%CI = − 0.01, 0.12; both *P <* 0.1). Nevertheless, residents put significantly less emphases on level 2 than JVSs (MD = − 0.76; 95%CI = − 0.14, − 0.02) and SVSs (MD = − 0.58; 95%CI = − 0.12, 0.00) did (both *P <* 0.05); nevertheless, both residents and JVSs put significantly more emphases on levels 4 and 5 than SVSs did.

**Table 5:** Multiple comparisons between statuses in the final Delphi survey round on five levels

	R vs. JVS			R vs. SVS			JVS vs. SVS		
Level	*MD*	*SE*	*95%CI*	*M*	*SE*	*95%CI*	*M*	*SE*	*95%CI*
**Lv. 1**	-0.04^†^	0.027	[-0.10, 0.00]	-0.02	0.027	[-0.08, 0.03]	0.023	0.021	[-0.02, 0.07]
**Lv. 2**	− 0.076^*^	0.030	[-0.14, -0.02]	− 0.058^*^	0.029	[-0.12, 0.00]	0.017	0.023	[-0.03, 0.06]
**Lv. 3**	0.017	0.032	[-0.05, 0.08]	0.057^†^	0.033	[-0.01, 0.12]	0.039	0.032	[-0.02, 0.1]
**Lv. 4**	-0.04	0.057	[-0.16, 0.07]	0.078^*^	0.030	[0.02, 0.14]	0.120^*^	0.050	[0.02, 0.22]
**Lv. 5**	0.055	0.034	[-0.01, 0.12]	0.144^**^	0.040	[0.06, 0.22]	0.088^*^	0.034	[0.02, 0.16]

Note. Adjustment for multiple comparisons: Least Significant Difference. † p-value < 0.10; * p-value < 0.05; ** p-value < 0.01

## Discussion

Implementing a milestone project outside the North America is challenging due to the difference in culture and healthcare system. Not only cultural diversity but also medical education are potential factors influencing PC [37]. Thus, cultural competence training could be exploited as a strategy to improve practitioners’ PC knowledge, attitudes, skills, and most importantly, competency [38]. The primary purpose of our study was to translate, co-produce, and indigenize the present ACGME milestones, a competency-based assessment, in anesthesiology for developing a well-informed draft for the later nation-wide consensus survey and investigating whether they are applicable for local practice. Four phenomena were thus noted: (a) indigenizing the ACGME anesthesiology milestones in Taiwan is feasible; (b) experienced anesthesiologists achieved consensus fast; (c) experience status affected the weight that anesthesiologists gave to milestone competencies; and (d) residents mirrored milestone competencies through participation.

### Indigenizing the ACGME anesthesiology milestones in Taiwan is feasible

This study’s primary finding showed that the ACGME anesthesiology milestones are highly feasible in Taiwan after indigenization. The expert committee and the Delphi process ensured the cross-cultural translation quality and validity. Most of the milestones are relevant to clinical practice and residents’ training, especially after the co-production process. Yet, one-seventh of them are difficult to be evaluated and might need further considerations in their implementation. Compared to a similar Delphi study, the cross-country differences in Anesthesiology are not as significant those in Emergency Medicine (14.7% versus 21%) [39]. The co-production model we adopted here helped us engage in learners’ voices, reflect democratic principles, and hopefully, contribute to a better educational outcome. We recruited trainees and junior specialists as representatives, which was also done in several recent Delphi studies [8, 40–42]. Although this indigenized document is only an introductory version, it is the first competency-based framework developed for the Taiwan anesthesia curriculum. Moreover, our preliminary results have later encouraged the Taiwan Society of Anesthesiologists (TSA) to set up a CBME taskforce and develop a nation-wide anesthesiology milestone for all training centers. This two-step approach, which started from a single-center development process followed by a national consensus, was also performed in other specialties [8, 9].

### Experience status affects the consensus and weight

The secondary finding of our study was that all three experience status groups, consisting of 37 anesthesiologists, reached a consensus on the six competencies domains at the end of the Delphi analysis with little difference (*P* = 0.059), even though some milestone items may not be directly applicable to the local context (***five stable nonconsensus items among residents and one stable nonconsensus item among JVSs***). Notably, we found gradients in consensus and nonconsensus items during round 1. SVSs, with more experience, demonstrated more consensus items (Table 2), and they attained complete consensus in round 2—which was compatible with the strong correlation between their rounds 2 and 3—in contrast to resident and JVS who achieved complete agreement only in round 3. This phenomenon probably resulted from the fact that experienced anesthesiologists (i.e., SVSs) tend to possess a higher quality of anesthesiology knowledge, skills, and communication ability than JVSs and residents do, which aids SVSs in cultivating competent anesthesiologists. The early consensus also indicated that these anesthesiology experts agreed regarding the anesthesiology training process.

As shown in Table 3, slightly decreasing trends were noted in the correlation coefficients between experience statuses, potentially because of different weights for the six competencies among the five levels and experience statuses (Tables 4 and 5). A possible factor underlying the domains having significant differences in weights based on the experience statuses is PC. As the first component of six domains, PC, is the most fundamental element in daily medical practice, highlighting the importance of patient-centered care as a mainstay approach to health care [43]. However, trainees, including medical students and residents, commonly overlook the importance of PC, mainly because trainees have a propensity to pursue higher achievements. For instance, in Taiwan, medical students ask to participate in clinical research to obtain more publications and apply for highly competitive specialties, such as anesthesiology. Residents’ academic activities are positively related to their clinical performance, and thus, they should be encouraged to utilize evidence-based medicine to enhance PC quality [44]. Moreover, PC should always remain prioritized during clinical practice and course training.

Our study also indicated that residents valued PROF more than the visiting staff. PROF represents the behaviors and attitudes toward patients, surgeons, and colleagues [45]. Multiple factors could explain our result: First, compared with surgeons or generalists, anesthesiologists rarely deal with patients who assume them to have a comprehensive perception of their chief complaint, present illness, medical history, and surgical procedure at their first preoperative meeting—where rapid evaluation of patient conditions is performed and perioperative reassurance is provided [45]. Second, coping with the surgical staff could be challenging for anesthesiology residents because some surgical staff members tend to refer to anesthesiologists as merely perioperative “consultants” rather than experts, even though anesthesiologists may have more thorough knowledge regarding patient status; as a result, residents, particularly those with less experience, find it difficult to simultaneously communicate with the surgical staff and provide good care to the patients during the perioperative period [45]. Taken together, all these factors tend to make younger residents consider themselves to have sufficient professionalism and anticipate fitting into surgical teams and treating patients well.

### Residents mirrored milestone competencies through participation

Another major highlight of our study was that although all nine residents had the highest number of nonconsensus items than JVS and SVS, the difference was insignificant during round 3 (*n* = 5) as stated above (*P* = 0.059). Moreover, the correlation coefficients between rounds 2 and 3 demonstrated a highly positive and significant correlation, with a trend of an increase in correlation in each round. This indicated that the residents (i.e., trainees) agreed with the contents of their training program. A study suggested that it is vital to include the trainees’ voices when developing evaluation tools for competency and performance because it is “likely to inform our understanding of whether and how assessments can serve the purpose of learning” [46, 47].

Another study on ACGME plastic surgery milestone evaluation reported that all residents, except for chief residents, significantly self-assessed higher than their attendings did; nevertheless, their evaluation results provided significant correlation coefficients. Thus, expectations for competency and performance standards differ between residents and their attendings [48]. The same effect was noted in an emergency department by Goldfam et al.; the residents there tended to overestimate their sub-competencies [49]. Notably, in the report above regarding plastic surgery milestones, [48] residents’ self-assessments throughout each year demonstrated the Dunning–Kruger effect [50]—a phenomenon related to the cognitive bias that causes people with low ability to overestimate their actual performance or competency. Therefore, letting residents know how they will be assessed and how they may rate themselves during courses could help them construct an image of a competent anesthesiologist, thus enhancing their confidence and easing their progress.

### Limitations and future directions

This study had several limitations. First, it was mainly conducted in two medical centers and two teaching hospitals in Taipei City, the capital of Taiwan, which has high resource availability and demand for health care services and related research and teaching. Second, the representative issue, such as whether residents or junior faculty members should be recruited into the panel as regarded as “experts”, might be challenged and criticized. Thus, our findings may not be generalizable to other situations, such as in areas with limited access to general health care (e.g., hospitals in remote areas). Future investigations that incorporate a nationally representative sample may be necessary for effective educational implementation.

## Conclusion

Most ACGME anesthesiology milestones are applicable in Taiwan. However, the five nonconsensus items noted in the current study warrant further detailed discussion during the implementation of the milestones. Moreover, our results contributed to the relevant perspectives of both senior staff members and residents. Our empirical results may guide medical educators when planning anesthesiology training curricula and raise their awareness regarding anesthesiology trainees’ learning process and understanding.

### Electronic supplementary material

Below is the link to the electronic supplementary material.

Supplementary Material 1

## Data Availability

Data described in the manuscript, code book, and analytic code will be made available upon request to the corresponding author Dr. Chien-Yu Chen (email: jc2jc@tmu.edu.tw).

## Abbreviations

ACGME: Accreditation Council for Graduate Medical Education
CBME: Competency-based medical education
CI: Confidence interval
ICS: Interpersonal and communication skills
IQR: Interquartile range
JVR: Junior visiting staff
MD: Mean difference
MK: Medical knowledge
PBLI: Practice-based learning and improvement
PC: Patient care
PROF: Professionalism
SBP: Systems-based practice
SD: Standard deviation
SE: Standard error
SVS: Senior visiting staff
